# Dyke-Davidoff-Masson Syndrome as a Predecessor of Recurrent Seizures in an Adult Male: A Report of a Rare Case

**DOI:** 10.7759/cureus.28838

**Published:** 2022-09-06

**Authors:** Sourya Acharya, Amol Andhale, Samarth Shukla, Pratik J Bhansali, Ruchita Kabra, Sunil Kumar

**Affiliations:** 1 Department of Medicine, Jawaharlal Nehru Medical College, Datta Meghe Institute of Medical Sciences (Deemed to be University), Wardha, IND; 2 Department of Medicine, Jawaharlal Nehru Medical College, Wardha, IND; 3 Department of Pathology, Jawaharlal Nehru Medical College, Datta Meghe Institute of Medical Sciences (Deemed to be University), Wardha, IND; 4 Department of Radiodiagnosis, Datta Meghe Institute of Medical Sciences, Wardha, IND; 5 Department of Internal Medicine, Datta Meghe Institute of Medical Sciences, Wardha, IND

**Keywords:** hemorrhage, hyperpneumatization, status epilepticus, sinus, hypoxia, congenital

## Abstract

Dyke-Davidoff-Masson syndrome (DDMS) is a rare condition that usually presents in early life with recurrent seizures. It can be congenital or can be acquired by perinatal hypoxia, infections, and intracranial hemorrhage. Its frequency remains unknown. It is usually diagnosed by neuroimaging. The classical neuroimaging features are unilateral cerebral hemiatrophy, volume loss, and hyperpneumatization of the sinus. We present the case of a 22-year-old male who presented with complex partial status epilepticus and had a history of recurrent seizures since he was six years old. The diagnosis of DDMS was made on neuroimaging.

## Introduction

Dyke-Davidoff-Masson syndrome (DDMS), also known as cerebral hemiatrophy, is a rare neurological condition that is only described in case reports [1-3]. Although it has classic neuroimaging findings, it is missed due to its complex presentation.

It was first described by Dyke, Davidoff, and Masson in nine patients who had hemiplegia. They described the neurologic features through changes seen in plain skull X-rays [1,2]. The characteristic clinical features include facial asymmetry, seizures, and contralateral hemiplegia which can occur in various combinations [1-3]. Signature neuroimaging findings include unilateral brain volume loss, ventriculomegaly, and compensatory bone hypertrophy resulting in cerebral hemiatrophy. In addition, there may be associated calvarial thickening and hyperpneumatization of frontal sinuses, which differentiates it from other conditions of hemiatrophy such as Rasmussen’s encephalitis and Sturge-Weber syndrome [3,4].

## Case presentation

A 23-year-old male presented to the emergency department with complex partial status epilepticus. The seizure was sudden in onset and was associated with a confused state and motor automatisms. It was managed conventionally by intravenous lorazepam and intravenous phenytoin. According to his mother, he was a preterm baby and was managed in the neonatal intensive care unit for hypoxic insult. In addition, he had a seizure disorder since he was six years old. He also had developmental delays in attaining milestones. There was no history of seizures in the family. He had been prescribed tab. sodium valproate 200 mg twice a day. The patient was taking tab. sodium valproate 200 mg twice a day till 14 years of age. Subsequently, he went to the hospital for a follow-up where computed tomography (CT) of the brain was done (scans not available). His dose was escalated after eight years at 14 years of age to 500 mg twice a day. However, he was non-compliant for four days when he presented with a seizure. Examination revealed mild right-sided hemiparesis with a power of 4/5, hyperreflexia, and extensor right plantar reflex. There was no facial atrophy. Cranial nerve and sensory examination were unremarkable.

**Figure 1:**
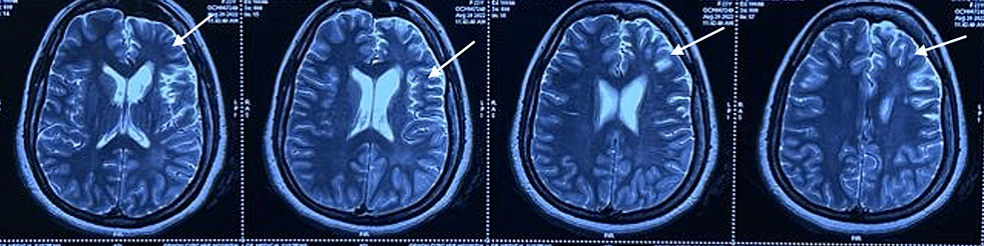
MRI of the brain: axial T2 images showing cerebral atrophy of the frontal and temporal lobe (white arrow). MRI: magnetic resonance imaging

**Figure 2:**
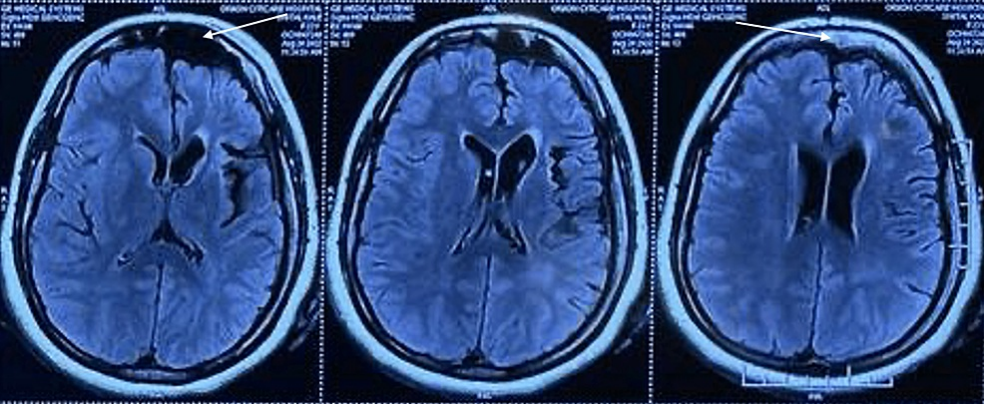
MRI of the brain: axial T2 FLAIR images showing calvarial thickening on the left side with hyperpneumatization of the frontal sinus (white arrow). MRI: magnetic resonance imaging; FLAIR: fluid-attenuated inversion recovery

**Figure 3:**
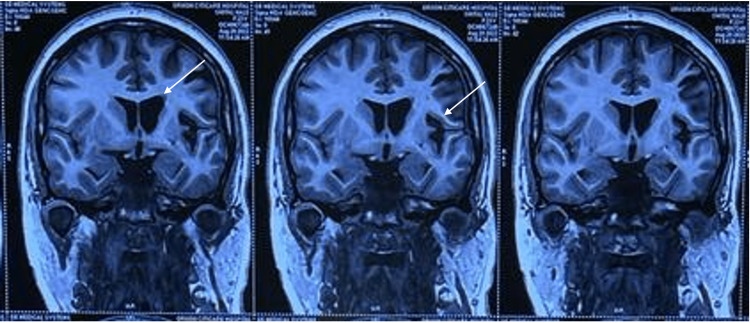
MRI of the brain: coronal FLAIR images showing dilated frontal horn of the left lateral ventricle (white arrow in the first image) with prominent left Sylvian fissure and sulcal spaces (white arrow in the middle image) secondary to left-sided cerebral atrophy. MRI: magnetic resonance imaging; FLAIR: fluid-attenuated inversion recovery

His laboratory investigations were normal. Magnetic resonance imaging (MRI) of the brain showed cerebral atrophy of the frontal and temporal lobe (Figure 1), calvarial thickening on the left side with hyperpneumatization of the frontal sinus (Figure 2), and dilated frontal horn of the left lateral ventricle with prominent left Sylvian fissure and sulcal spaces secondary to left-sided cerebral atrophy (Figure 3). These features were suggestive of DDMS. Electroencephalogram revealed continuous epileptogenic discharges in the form of lateralized sharp waves and spikes.

He was discharged on sodium valproate 500 bid and was advised for regular follow-ups.

## Discussion

Dyke, Davidoff, and Masson described DDMS in 1933 [4]. Nine patients with hemiparesis, facial asymmetry, seizures, intellectual disability, and findings of pneumatoencephalographic changes on skull radiography presented with DDMS, a rare neurological condition named after the three doctors who first described it in 1933 [4]. Although the exact cause of DDMS remains unknown, previous studies have linked the disorder to a brain injury that occurred either in utero or during early childhood as a result of trauma, ischemia, hemorrhage, or infection [5]. This condition is known as the congenital subtype and manifests in infants.

The clinical presentations of DDMS include seizures, mental retardation, contralateral hemiparesis, and facial asymmetry. Our patient had recurrent seizures since childhood and examination revealed hemiparesis. History revealed that he was a preterm baby with a hypoxic insult at birth. DDMS was diagnosed by MRI of the brain. Initially, he complained of headaches, followed by episodic complex partial seizures. This rare condition can be missed if not diligently sought for because these characteristic imaging findings become gradually evident with increasing age [5]. Especially the calvarial thickening suggests cerebral damage in the intrauterine period [6-8].

The radiological results of CT scans and MRIs are crucial in making the diagnosis and determining the severity of the ailment, in addition to the clinical presentation. These results can differ from patient to patient, although they frequently include lateral ventricular enlargement, prominent sulci, and atrophy of the cerebral hemisphere next to the lesion. The differential diagnoses of DDMS include Rasmussen encephalitis and Sturge-Weber syndrome [3,4]. Sturge-Weber syndrome is also associated with the port wine stain on the face and Rasmussen encephalitis does not show calvarial thickening on neuroimaging. Unilateral cerebral atrophy with compensatory hypertrophy of the skull bones and hyperpneumatization of air sinuses such as the frontal sinus are characteristic signs of DDMS [1,9].

The DDMS management strategy focuses on symptom relief. Depending on the needs of the patient, various modalities may be used, including anticonvulsant drugs for seizure management, physical therapy for neurological rehabilitation, and speech therapy. Hemispherectomy is recommended in situations of hemiplegia with uncontrollable incapacitating seizures, with a reported 85% success rate [9]. The prognosis is poor for patients with repeated and persistent seizures and hemiparesis that start before the age of two [10]. Because there are no established standards for the care of seizures in these patients, our patient was started on valproic acid to avoid repeated seizures [11].

## Conclusions

DDMS is a syndrome that is usually misdiagnosed. It presents with recurrent seizures since childhood along with focal neurologic deficits. Cerebral hemiatrophy is the classic imaging finding. Though cerebral hemiatrophy is also seen in conditions such as Rasmussen encephalitis and Sturge-Weber syndrome, the hallmark neuroimaging finding that differentiates it is calvarial thickening and hyperpneumatization of the frontal sinus. Thorough knowledge of the clinical presentation, associated risk factors, such as birth asphyxia, intracranial hemorrhage, infections, and imaging features are essential elements to suspect and diagnose DDMS.
